# The relationship between dopamine receptor D1 and cognitive performance

**DOI:** 10.1038/npjschz.2014.2

**Published:** 2015-03-04

**Authors:** Jonathan Tsang, John F Fullard, Stella G Giakoumaki, Pavel Katsel, Pavel Katsel, Vasiliki Eirini Karagiorga, Tiffany A Greenwood, David L Braff, Larry J Siever, Panos Bitsios, Vahram Haroutunian, Panos Roussos

**Affiliations:** 1 Icahn School of Medicine at Mount Sinai, New York, NY, USA; 2 Department of Psychiatry, New York, NY, USA; 3 Friedman Brain Institute, New York, NY, USA; 4 Department of Psychology, University of Crete, Rethymno, Crete, Greece; 5 Department of Psychiatry, Icahn School of Medicine at Mount Sinai, Athens, Greece; 6 Department of Psychiatry, University of California, San Diego, CA, USA; 7 VISN-22 Mental Illness Research, Education, and Clinical Center, VA San Diego Healthcare System, San Diego, CA, USA; 8 James J. Peters VA Medical Center, Mental Illness Research Education and Clinical Center (MIRECC), 130 West Kingsbridge Road, Bronx, NY, USA; 9 Department of Psychiatry, Faculty of Medicine, University of Crete, Heraklion, Crete, Greece; 10 Computational Medicine Laboratory, Institute of Computer Science at FORTH, Heraklion, Greece; 11 Department of Genetics and Genomic Sciences, New York, NY, USA; 12 Institute for Genomics and Multiscale Biology, New York, NY, USA

## Abstract

**Background:**

Cognitive impairment cuts across traditional diagnostic boundaries and is one of the most typical symptoms in various psychiatric and neurobiological disorders.

**Aims:**

The objective of this study was to examine the genetic association between 94 candidate genes, including receptors and enzymes that participate in neurotransmission, with measures of cognition.

**Methods:**

The Clinical Dementia Rating (CDR), a global measure of cognition, and genotypes derived from a custom array of 1,536 single-nucleotide polymorphisms (SNPs) in 94 genes were available for a large postmortem cohort of Caucasian cases with Alzheimer’s disease (AD), schizophrenia and controls (*n*=727). A cohort of healthy young males (*n*=1,493) originating from the LOGOS project (Learning On Genetics Of Schizophrenia Spectrum) profiled across multiple cognitive domains was available for targeted SNP genotyping. Gene expression was quantified in the superior temporal gyrus of control samples (*n*=109). The regulatory effect on transcriptional activity was assessed using the luciferase reporter system.

**Results:**

The rs5326-A allele at the promoter region of dopamine receptor D1 (*DRD1*) locus was associated with: (i) poorer cognition (higher CDR) in the postmortem cohort (*P*=9.325×10^−4^); (ii) worse cognitive performance relevant to strategic planning in the LOGOS cohort (*P*=0.008); (iii) lower *DRD1* gene expression in the superior temporal gyrus of controls (*P*=0.038); and (iv) decreased transcriptional activity in human neuroblastoma (SH-SY5Y) cells (*P*=0.026).

**Conclusions:**

An interdisciplinary approach combining genetics with cognitive and molecular neuroscience provided a possible mechanistic link among *DRD1* and alterations in cognitive performance.

## Introduction

Cognitive impairment is one of the most prevalent symptoms associated with various psychiatric and neurobiological disorders, including schizophrenia and Alzheimer’s disease (AD).^1^ Independent of the diverse etiopathogenetic mechanisms associated with different diseases, multiple studies have demonstrated the important modulatory role of neurotransmission on cognitive function.^2^ Therefore, the pathological alterations of neurotransmitter systems might contribute to cognitive impairment and/or may account for the progression of cognitive decline.

The Clinical Dementia Rating (CDR) is an interviewer-based quantitative variable that assesses a person’s cognitive and functional performance in six areas: memory, orientation, judgment and problem solving, community affairs, home and hobbies, and personal care.^3^ On the basis of CDR classification, subjects are grouped as no cognitive deficits (CDR=0), questionable dementia (CDR=0.5, sometimes classified and mild cognitive impairment), mild dementia (CDR=1.0), moderate dementia (CDR=2.0), and severe-to-terminal dementia (CDR=3.0–5.0). CDR is a global measure of cognition, which is abnormal across many disorders, including schizophrenia^4^ and AD.^5^

The purpose of this study was to examine the genetic association between 94 candidate genes, including receptors and enzymes that participate in neurotransmission, and CDR in a large postmortem cohort of cases with AD, schizophrenia, and controls. Targeted genotyping was done using the Consortium on the Genetics of Schizophrenia (COGS) single-nucleotide polymorphism (SNP) array. This is a custom array that was used previously to examine the genetic association of candidate genes with multiple schizophrenia endophenotypes.^6^ It includes 1,536 SNPs in 94 genes, selected on the basis of previous evidence for genetic association with schizophrenia or cognition and molecular pathways discovered through gene expression, brain imaging, or model organism studies. The majority of candidate genes are involved in synaptic transmission signaling, including glutamate, GABA, dopamine, and serotonin. In an attempt to provide a proof-of-principal confirmation of the most significant association in the postmortem study (a SNP is the promoter region of the dopamine D1 receptor), the association of this SNP with cognitive function was evaluated in an independent living young adult cohort (LOGOS sample). To gain additional functional support for our findings, we then tested the regulatory potential of the most significant locus in human brain gene expression and *in vitro* luciferase experiments.

## Materials and methods

### Subjects

#### Brain tissue samples

Brain tissue specimens were obtained from the Icahn School of Medicine at Mount Sinai and the JJ Peters VA Medical Center MIRECC Brain Bank. The precise tissue handling procedures have been described in detail previously.^7–10^ All antemortem neuropsychological, diagnostic, and autopsy protocols were approved by the Icahn School of Medicine at Mount Sinai and other relevant Institutional Review Boards. Each sample has been extensively characterized on the basis of clinical and neuropathological criteria in diagnostically relevant (Consortium to Establish a Registry for Alzheimer’s Disease) brain regions,^11^ including the: (i) CDR;^3,12^ (ii) density of neuritic plaques; and (iii) distribution of neurofibrillary tangle pathology using Braak neuropathology staging.^13^ For more details, see Supplementary Methods. The CDR was used as the primary measure of dementia severity. A multi-step consensus-dependent approach was applied to the assignment of CDR scores based on cognitive and functional status during the last 6 months of life, as described previously.^9,10^ Medical histories and records were reviewed for possible indirect causes of cognitive impairments such as renal disease, delirium, sepsis, etc. When available, longitudinal neuropsychological assessment results were also considered in deriving the final consensus CDR score.

#### Healthy individuals

A total of *N*=1,493 healthy males (age mean±s.d.: 22.05±3.45, range: 18–30 years), who were recruited for the LOGOS (Learning on Genetics of Schizophrenia) study, underwent cognitive assessment and consented to providing DNA. Detailed description of the LOGOS study and the inclusion and exclusion criteria has been published previously.^14–19^ Briefly, a review of the participants’ medical history was taken, and the Mini-International Neuropsychiatric Interview,^20^ urine toxicology, and IQ testing with the Raven’s Progressive Matrices^21^ were performed. The study was conducted according to the principles expressed in the Declaration of Helsinki and was approved by the Ethics Committee of the University of Crete, the Executive Army Bureau, and the Bureau for the Protection of Personal Data of the Greek State. All subjects were administered the Wisconsin Card Sorting Test^22^ assessing set-shifting and rule learning abilities, the Word Lists task^23^ assessing verbal learning and memory, the Iowa Gambling task^24^ assessing emotional decision making and the N-back Sequential Letter task^25^ assessing working memory. They were also administered selected tasks from the Cambridge Neuropsychological Test Automated Battery: spatial working memory and strategy formation were assessed with the Spatial Working Memory task,^26^ sustained attention and vigilance were assessed with the Rapid Visual Information Processing task,^27^ and planning and problem solving were assessed with the Stockings of Cambridge.^26^ For more details see Supplementary Methods.

### DNA extraction and genotyping

#### Brain tissue samples

Samples of DNA from the postmortem cohort were extracted from the superior temporal gyrus using the Genomic DNA-Tissue MiniPrep kit (Zymo Research, Irvine, CA, USA). Genotyping was performed blind to phenotype measures using the COGS SNP array.

#### Healthy individuals

DNA from the LOGOS cohort was extracted from blood or cheek swab samples, using the QIAamp DNA Blood Mini Kit (Qiagen, Hilden, Germany). Genotyping of *DRD1* rs5326 was performed blind to phenotype measures by LGC Genomics (Herts, UK) (http://www.lgcgenomics.com/) with a competitive, allele-specific PCR system. The call rate for rs5326 was 98.46% and genotype frequencies were distributed in accordance with Hardy–Weinberg equilibrium (*χ*^*2*^=0.35; *P*=0.56). Genotyping quality control for each SNP was performed in 50 randomly selected samples by duplicate checking (rate of concordance in duplicates >99%). All the subjects were of Caucasian ancestry on the basis of self-report, which was confirmed for a subset of the cohort (833 out of 840 subjects) on the basis of EIGENSOFT analysis^28,29^ of genome-wide genotyping SNP profiling with the Illumina HumanOmniExpress BeadChip^19^ (San Diego, CA, USA). On the basis of these data, the self-report identification of the Caucasian ancestry is 99.2% (833/840) reliable, which makes genetic inhomogeneity of the tested population unlikely.

### RNA extraction and real-time quantitative PCR

Total RNA was extracted from 50 mg of frozen tissue prepared from the superior temporal gyrus, as described in detail elsewhere.^18,19,30^ Brain specimens from 109 controls samples with Caucasian ancestry were used. The messenger RNA (mRNA) level of *DRD1* was measured by quantitative PCR using TaqMan probes and primer sets (Applied Biosystems, Foster City, CA, USA). TaqMan probe identification numbers are listed in Supplementary Table 1. For relative quantification of mRNA expression, geometric means of the expression of three housekeeping genes (*GUSB*, *PPIA*, and *RPLPO*) were calculated using the standard curve method.

### Transient transfection and luciferase assays

Luciferase reporter plasmids were constructed by cloning the regulatory sequence containing rs5326 into the pGL4.11 vector (Promega, Madison, WI, USA) upstream of *luc2P*. The regulatory sequence of rs5326 G and A alleles±250 bp upstream/downstream (hg19; chr5:174,869,946–174,870,446) was introduced at the 5′ and 3′ using *KpnI* and *XhoI*. We sequenced the inserted portions of the constructs to verify the nucleic acid sequences and the location of the SNP. Human neuroblastoma (SH-SY5Y) cells were transfected with each reporter vector (450 ng) and the Renilla luciferase expression vector pRL-TK (200 ng, Promega) using Lipofectamine 2000 (3 μl:1 μg Lipofectamine: DNA) (Invitrogen, Grand Island, NY, USA) in 200 μl Opti-MEM (Invitrogen) in 12-well plates. SH-SY5Y were grown in 1 ml of 1:1 mixture of DMEM and Ham’s F12 medium supplemented with 10% fetal bovine serum. The media was not changed after the addition of the transfection reagents. Twenty-four hours after transfection, cells were collected and lysed by the addition of 250 μl of Passive Lysis Buffer (Promega). The luciferase activity in the cell lysates was determined using the Dual Luciferase Reporter System (Promega) in quadruplicates. The experiment was repeated five times as independent experiments. Firefly luciferase activities were normalized to that of Renilla luciferase and expression relative to the empty pGL4.11 vector was noted.

### Statistical analysis

Demographics data were compared among groups using Kruskal–Wallis (continuous, nonparametric variables), analysis of variance (continuous, parametric variables) or chi-square (categorical variables). All statistics were two-tailed, and significance was set at *P*<0.05.

#### Brain tissue samples

The preprocessing of SNP data and genetic analysis was performed with Plink (Version 1.07).^31^ More specifically, individuals were removed if they were outliers with respect to estimated heterozygosity (more than 3 s.d.) or had missing SNPs >5%. SNPs were removed if: missing genotype rate >5%; Hardy–Weinberg equilibrium *P*<10^−3^, minor allele frequency <5%. For any pair of subjects with cryptic relatedness (pi-hat >0.125 in PLINK, the sample with the lower call rate was eliminated. After QC, we had genotype profiling for 1,093 SNPs in 727 individuals (148 controls, 349 cases with AD, and 230 cases with schizophrenia) with total genotyping rate 99.9%. All the subjects were of Caucasian ancestry on the basis of self-report, which was confirmed for a subset of the cohort (310 out of 318 subjects or 97.5%) on the basis of EIGENSOFT analysis^28,29^ of genome-wide genotyping SNP profiling using the Affymetrix SNP 6.0 array (Santa Clara, CA, USA). We used a linear regression model to examine the association of CDR and genotypes across diagnostic groups:

$$\mathrm{CDR}=\beta_{0}+\beta_{G}G+\beta_{\mathrm{Sex}}\mathrm{Sex}+\beta_{\mathrm{Age}}\mathrm{Age}+\beta_{\mathrm{Scz}}\mathrm{Scz}+\beta_{\mathrm{AD}}\mathrm{AD}+\beta_{G\times \mathrm{Scz}}\mathrm{GScz}+\beta_{G\times \mathrm{AD}}\mathrm{GAD},$$

where *β*_G_ is the parameter of interest quantifying the association between a genotype G and the mean of CDR controlling for sex, age, disease status—schizophrenia (Scz) and AD, and disease by genotype interaction for schizophrenia (G×Scz) and AD (G×AD). To correct for multiple testing and reduce the probability of type I error, empirical *P* values (*P*_emp_) were estimated on the basis of 100,000 permutations.

#### Healthy individuals

For the sake of data reduction and variables’ classification we subjected the outcome variables from the cognitive tasks to principal component analysis (PCA). For PCA, the varimax rotation method was used and components with eigenvalues >1 and factor loadings >0.5 were accepted. Analysis of covariance was used for comparison of cognitive performance among genotype groups with age and smoking status as covariates. *P* values were Bonferroni corrected by dividing 0.05 by the number of tested cognitive domains.

#### Gene expression and luciferase experiments

Pearson correlations were performed to examine the relation of potential confounds (age, gender, postmortem interval, RNA integrity number) with *DRD1* gene expression. We used a linear regression model to examine the association of *DRD1* gene expression and genotypes using RNA integrity number and age as covariates. Independent *t*-test was used for the analysis of the luciferase *in vitro* experiments.

## Results

### Association of COGS SNPs with CDR in postmortem cohort

**Figure 1:**
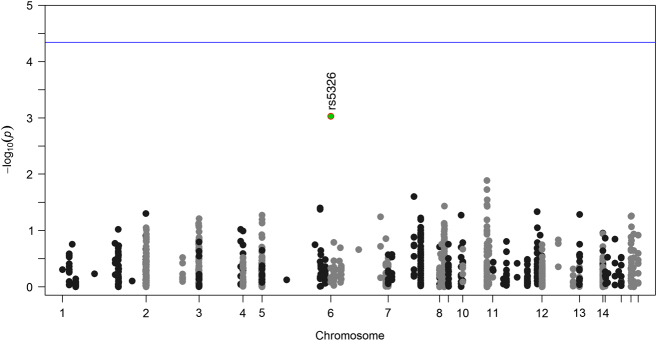
Genetic association analysis of 1,093 COGS SNPs and CDR in 727 individuals. Each SNP association is represented by a dot. The blue line represents the Bonferroni multiple testing correction threshold (*P*<4.58×10^−5^). The strongest association was observed for rs5326.

The association of COGS SNPs with CDR was examined across all groups (controls and cases with schizophrenia and AD) using a linear regression model. The strongest association was observed for rs5326 (*β*=0.74; *P*=9.325×10^−4^), which survived corrections for multiple testing (*P*_emp_=0.004). Figure 1 and Supplementary Table 2 shows the association between CDR and COGS SNPs. Separate analysis of CDR in diagnostic groups, showed that rs5326 had the strongest association in controls (*β*=0.71; *P*=9.78×10^−5^) and cases with AD (*β*=0.86; *P*=2.81×10^−4^), while ranked third in cases with schizophrenia (*β*=0.61; *P*=8.05×10^−4^). The rs5326 is positioned in the promoter region of dopamine receptor D1 (*DRD1*).

### Association of DRD1 rs5326 with cognitive performance in LOGOS

**Figure 2:**
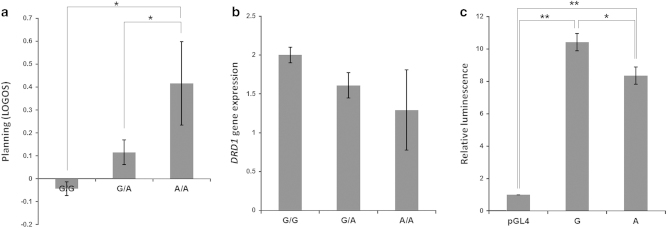
Association of *DRD1* rs5326 with (**a**) cognitive performance (Strategic planning) in the LOGOS cohort (F(2,1465)=5.847; *η*^*2*^=0.008; *P*=0.003), (**b**) *DRD1* gene expression (*R*^*2*^=0.124; *β*=−0.353; *P*=0.038) and (**c**) relative luciferase activity in human neuroblastoma (SH-SY5Y) cells (*P*=0.026). Error bars represent s.e.m. *P<0.05; **P<0.005.

There was a significant difference among genotype groups (*P*<0.05) for age and smoking history (Supplementary Table 3) and both variables were added as covariates in the analysis of covariance. Eigenvalues for nine cognitive domains were derived on the basis of PCA of 22 outcome variables from seven cognitive tasks (Supplementary Table 4). The rs5326-A allele was associated with increased latency in a cognitive domain relevant to strategic planning (F(2,1465)=5.847; *η*^*2*^=0.008; *P*=0.003) based on analysis of covariance comparison using age and smoking status as covariates (Figure 2a). *Post hoc* comparisons with the Bonferroni test correction revealed that the G/G group had lower latencies for strategic planning than the G/A (*P*=0.033) or the A/A (*P*=0.039) groups. Higher strategic planning latencies indicate increased mean initial and subsequent thinking time during the Stockings of Cambridge task.

### Association of rs5326 with expression of *DRD1* mRNA in human brain and transcriptional activity

The rs5326-A allele was significantly associated with decreased *DRD1* gene expression (*R*^*2*^=0.124; *β*=−0.353; *P*=0.038; Figure 2b). RIN and age were included as covariates in the linear regression model, as there were nominally significant differences (*P*<0.1) among genotype groups (Supplementary Table 5) and RIN was also positively correlated with *DRD1* gene expression (Pearson’s *r*=0.271; *P*=0.004). The association of rs5326 with *DRD1* gene expression remain significant in permutation analysis of 100,000 random sets of genotypes relative to *DRD1* gene expression (empirical *P*=0.008).

The effect of rs5326 on transcriptional activity was examined in *in vitro* experiments. Compared with the reference rs5326 G allele, the A variant was associated with decreased transcriptional activity in human neuroblastoma (SH-SY5Y) cells (20%; *P*=0.026; Figure 2c).

## Discussion

This study applied an interdisciplinary approach combining genetics with cognitive and molecular neuroscience and provided a possible mechanistic link between *DRD1* gene variants and expression and cognitive performance. We initially performed a targeted genetic analysis in a clinically and neuropathologically characterized postmortem cohort, using an informative panel of SNPs positioned within genes that affect and regulate synaptic transmission (COGS SNP array).^6^ From the 1,093 SNPs selected for association testing, rs5326 showed the strongest effect on CDR, a global measurement of cognition. For further exploration of the genetic association with cognition, we examined the association of rs5326 across nine different cognitive domains in the LOGOS cohort. We found a significant association of rs5326 with a PCA factor best described as strategic planning. Because the rs5326 is positioned at the promoter region of *DRD1*, we examined the regulatory effect of this variant on transcriptional activity. The risk rs5326 allele was associated with decreased *DRD1* gene expression and luciferase transcriptional activity. These results replicate and extend previous findings on the association of the *DRD1* with neurocognitive function.

The role of dopaminergic neurotransmission in modulating neurocognitive function is well established.^32^ The dopamine D1 receptor is abundantly expressed in the prefrontal cortex (PFC).^33^ Infusion of D1 receptor antagonists into PFC in nonhuman primates induces deficits in working memory task.^34^ In human subjects, administration of a mixed D1/D2 agonist facilitates working memory, while the selective D2 agonist has no effect, indicating the important modulatory role of dopamine D1 receptor on neurocognitive function.^35^ This has led to the development of hypotheses that D1 agonists represent a promising approach to the treatment of cognitive impairment.^36^

Multiple studies have provided evidence supporting an inverted U-shaped model between PFC dopaminergic signaling and performance in PFC-dependent cognitive tasks.^37,38^ The relationship of genetic variants within catechol-O-methyltransferase gene and cognitive function is well established.^39–43^ It has been demonstrated that tolcapone, a selective inhibitor of the catechol-O-methyltransferase enzyme, improves cognitive performance only in individuals that have lower capacity of PFC DA neurotransmission system, stratified according to their genetic background.^39–41^ We found that the rs5326 is a functional SNP with a potential important role in ‘placing’ human subjects on the low end of the inverted U-shaped curve, contributing to the impaired cognitive function and predicting the beneficial effect of D1 agonists on cognition.

The data presented here provide direct evidence for the association of low-levels of cortical *DRD1* mRNA gene expression, which is determined by genetic factors, with neurocognitive performance. The D1 receptor is affected by AD^44,45^ and schizophrenia,^46,47^ and decreases with age.^48,49^ Here, we applied a statistical model that does consider the confound effect of disease status and age. In addition, the strong effect of rs5326 on CDR was also observed in control brain samples, while a similar effect on cognitive function was observed in healthy young males. All the above indicate that the association of CDR with rs5326 is not limited only in cohorts with low cognitive reserve. One limitation is that the LOGOS cohort includes only healthy young males and caution must be exercised when considering our findings in the context of the general population.

A previous study did not find a significant association among rs5326 and *DRD1* gene expression.^47^ The inconsistency of our results might be secondary to differences in the characteristics of the human postmortem cohort, including younger controls (mean age was 40, where variation in cognitive performance may be less) and mixed ethnic population. One limitation of our study is that we did not explore the effect of the rs5326 on protein abundance and D1 receptor availability. While ~40% of the variation in protein concentration can be explained by knowledge of mRNA abundances, additional mechanisms such as posttranscriptional, translational regulation, and degradation rates, acting through miRNAs may fine tune protein abundances.^50^ Thus, predicting D1 receptor availability and abundance at the surface of neurons on the basis of gene expression data is challenging and possibly misleading. Another limitation is that the gene expression profiling was conducted in a single brain region (superior temporal gyrus). A previous study found that among 15 cortical regions, the superior temporal gyrus shows the highest transcriptional vulnerability associated with CDR.^7^ However, other regions, such as dorsolateral prefrontal cortex, mediate cognitive functions normally associated with DRD1 function (e.g., planning, working memory, etc.) and should be further examined in future gene expression studies.

Overall, the data presented in this study provide a mechanistic link between *DRD1* availability and cognitive performance across diagnostic groups. Additional support for the potential of targeting neurocognitive deficits through D1 receptor agonists is provided. Future studies should explore the effect of D1 receptor agonists on improving cognitive function in an inverted U-shaped dependent manner, taking into consideration the *DRD1* genetic background.
