# Supplementary material for: The relationship between dopamine receptor D1 and cognitive performance
Source: NPJ Schizophr. 2015 Mar 4;1:14002–. doi: 10.1038/npjschz.2014.2 (PMC4849437; doi:10.1038/npjschz.2014.2)
Supplement: Supplementary Information [file npjschz20142-s1.doc]

**Methods**

**Subjects**

**Brain tissue samples**

Brain tissue specimens were derived from the Icahn School of Medicine at Mount Sinai and the JJ Peters VA Medical Center MIRECC Brain Bank. The precise tissue handling procedures have been described in detail previously[^1-4^](#_ENREF_1). Tissue donors were persons who had been residents of area nursing homes, independent and assisted living facilities and the community at large many of whom were participants in longitudinal studies of aging and dementia. All subjects died of natural causes without a history of illicit drug use. The predominant causes of death were cardiovascular disease and myocardial infarction, cancer, septicemia and bronchopneumonia. All antemortem neuropsychological, diagnostic and autopsy protocols were approved by the Icahn School of Medicine at Mount Sinai and other relevant Institutional Review Boards. Each sample has been extensively characterized, based on clinical and neuropathological criteria in diagnostically relevant [Consortium to Establish a Registry for Alzheimer's Disease (CERAD) –defined] brain regions[^5^](#_ENREF_5), including the: (i) clinical dementia rating (CDR); (ii) neuritic plaques (NP) density; and (iii) distribution of neurofibrillary tangle (NFT) pathology using Braak neuropathology staging[^8^](#_ENREF_8). CERAD criteria were used to group cases into normal brain (CERAD-1), definite AD (CERAD-2), probable AD (CERAD-3) or possible AD (CERAD-4). Donors with significant non-AD associated neuropathology, such as infarcts, Lewy body disease and other neuropathologies, were excluded as described previously[^9^](#_ENREF_9).

The CDR was used as the primary measure of dementia severity. A multi-step consensus-dependent approach was applied to the assignment of CDR scores based on cognitive and functional status during the last 6 months of life as described previously. When available, longitudinal neuropsychological assessment results were also considered in deriving the final consensus CDR score. Neuropathological assessments were performed on the right hemisphere and consisted of examining representative blocks from the superior and middle frontal gyrus, orbital cortex, basal ganglia with basal forebrain, amygdala, hippocampus (rostral and caudal levels with adjacent parahippocampal and inferior temporal cortex), superior temporal gyrus, parietal cortex (angular gyrus), calcarine cortex, hypothalamus with mammillary bodies, thalamus, midbrain, pons, medulla, cerebellar vermis, and lateral cerebellar hemisphere according to the CERAD protocol. Sections from paraffin embedded blocks were variably stained with hematoxylin and eosin, modified Bielschowski, modified thioflavin S, and anti-β amyloid (clone 6F/3D, Dako Corp., CA), anti-tau (AD2, gift of Dr. A. Delacourte, Lille, France) and anti-ubiquitin (Daka Corp. Carpinteria, CA staining of representative) as required. All neuropathology data regarding the extent and distribution of neuropathologic lesions were collected in a blinded fashion relative to the subject's dementia status. The average of NP density measures in the cerebral cortex, as well as quantitative data in the middle frontal gyrus (Brodmann area 9), orbital frontal cortex (Brodmann area 45/47), superior temporal gyrus (Brodmann area 21/22), inferior parietal cortex (Brodmann area 39) and calcarine cortex (Brodmann area 17) were collected as described[^3^](#_ENREF_3). Each case was assigned a Braak AD-staging score for progression of neurofibrillary neuropathology in seven distinct brain regions: hippocampus, entorhinal cortex (Brodmann area 28/34), amygdala, superior temporal gyrus (Brodmann area 21/22), middle frontal gyrus (Brodmann area 9), inferior parietal cortex (Brodmann area 7) and calcarine cortex (Brodmann area 17). Based on recruitment and inclusion-exclusion criteria, preliminary analysis of the neuropathology data set revealed that Lewy bodies were so infrequently encountered as to be uninformative. Similarly, NPs and NFTs in brainstem nuclei were evenly and sparsely distributed and did not differentiate between AD and non-AD and dementia vs. non-dementia cases. These brain regions were therefore excluded from further analyses.

**Neurocognitive tasks**

*Wisconsin Card Sorting Test*[^12^](#_ENREF_12). A computerized version was used. The task consisted of four stimulus cards that varied along three dimensions (color, shape, and number). Participants were asked to match the cards in the deck with one stimulus card and feedback was provided after each selection. Once six consecutive cards were categorized correctly, the sorting principle changed. We used a modified version of the task, as suggested by Nelson[^12^](#_ENREF_12) so that the examiner tells the subject when the matching principle changes, thus making clearer what is being measured by the test. Outcome variables were the total number of categories achieved and total errors.

*Word Lists task*[^13^](#_ENREF_13): A list of 12 words was read, and subjects were asked to recall the words in any order (immediate recall); this procedure was repeated four times. After Trial 4, an interference trial with a new list occurred, and subjects were subsequently asked to recall as many words as possible from the first list (short-delay recall). Thirty minutes later, subjects were asked to recall the words from the first list again (long-delay recall). The test finished with a recognition memory trial: a list of words was read, and subjects were asked to identify the words included in the first list (recognition). Outcome variables were the number of correct responses per recall condition (immediate-, short delay-, long delay-recall) and intrusion errors (words identified that were not included in the list).

*Iowa Gambling Task*[^14^](#_ENREF_14): This is a simulated gambling task administered on a computer. Participants are given € 2000 in computer money and are instructed to lose as little or make as much money as possible by selecting cards (one at a time) from four decks (A–D) displayed on the screen. They are advised that each card has a different monetary value but no other information is given. Cards in decks A and B are associated with high monetary rewards but also high penalties (monetary loses) while those in decks C and D have lower rewards but also lower penalties. Participants learn the monetary value of each card after they have selected it. Across 100 trials, more choices from the decks C and D lead to a net gain while choosing from the other two decks results in greater loss. Outcome variables were the total money won and the total numbers of cards selected from advantageous decks C and D minus the total numbers of cards selected from (“risky”) decks A and B (CD−AB difference), with a higher score indicating superior performance.

*N-Back Sequential Letter Task*[^15^](#_ENREF_15): The task consisted of four conditions (0-, 1-, 2-, and 3-back) where subjects were asked to respond by a button press when they saw a target letter (letter “X” for 0-back and any letter that was identical to the one presented in the preceding 1, 2, or 3 trials, respectively) on the computer screen. The outcome variables were the number of correct responses in each condition.

*Cambridge Neuropsychological Test Automated Battery (CANTAB) tasks*: We used three subtests of the CANTAB[^16^](#_ENREF_16) namely, Spatial Working memory (SWM), Stocking of Cambridge (SoC) and Rapid Visual Information Processing (RVIP). These are non-verbal tests which were administered with the aid of a touch-sensitive screen and/or a response key.

The SWM tests spatial working memory and strategy[^16^](#_ENREF_16). Subjects are required to search through an increasing number (four, six, and eight) of boxes randomly arranged on the screen, until they find a single token that, at any one time, is hidden in one of the boxes. The key instruction is that once a token has been found within a particular box, then that box should never be used again to hide a token. On each trial, every box is used once to hide a token such that the total number of tokens to be found corresponded to the number of boxes on the screen. Errors are scored according to the number of occasions on which a subject returns to open a box in which a token has already been found. An efficient strategy for completing this task is to follow a predetermined search sequence, beginning with a particular box and then returning to start each new sequence with that same box as soon as a token has been found. The extent to which this repetitive searching pattern is used as a strategy for approaching the problem is estimated from the number of search sequences starting with the same box, within each of the more difficult 6- and 8-box problems. The total of these scores provides a single measure of strategy for each subject, with a high score (many sequences beginning with a different box) representing low use of the strategy and vice versa. Outcome variables were the strategy score, total between errors (defined as times the subject revisits a box in which a token has already been found) and total within errors (defined as the number of times the subject revisits a box already found to be empty during the same search).

The SoC is a modified, computerized version of the Tower of London[^16^](#_ENREF_16). Subjects are asked to compare two different arrangements of “balls” in “socks” (one presented on the top half of the screen, the other on the bottom) and rearrange, in the minimum possible number of moves, the balls in the lower half of the screen such that their positions match the target arrangement in the upper half. The test presents the subject with easy 2- and 3-move and harder 4- and 5-move problems. Subjects are asked to plan the complete sequence of moves required to solve the problem prior to their first move. Outcome variables were the number problems solved in minimum moves, mean number of moves, mean initial thinking time (ITT; i.e. the time prior to execution of the first move) and subsequent thinking time (STT; i.e. the time for the execution of all subsequent moves).

The RVIP tests sustained attention and vigilance[^17^](#_ENREF_17). Subjects are asked to detect consecutive target sequences of digits presented at the rate of 100 digits per minute for 4 min and responses are registered by a button press. Outcome variables were the number of correct responses and the number of stimuli correctly rejected.

**References**

1. Haroutunian, V., Katsel, P. & Schmeidler, J. Transcriptional vulnerability of brain regions in Alzheimer's disease and dementia. *Neurobiol Aging* **30**, 561-73 (2009).

2. Davis, K.L. *et al.* Cholinergic markers in elderly patients with early signs of Alzheimer disease. *JAMA* **281**, 1401-6 (1999).

3. Haroutunian, V. *et al.* Regional distribution of neuritic plaques in the nondemented elderly and subjects with very mild Alzheimer disease. *Arch Neurol* **55**, 1185-91 (1998).

4. Haroutunian, V. *et al.* Neurofibrillary tangles in nondemented elderly subjects and mild Alzheimer disease. *Arch Neurol* **56**, 713-8 (1999).

5. Mirra, S.S. *et al.* The Consortium to Establish a Registry for Alzheimer's Disease (CERAD). Part II. Standardization of the neuropathologic assessment of Alzheimer's disease. *Neurology* **41**, 479-86 (1991).

6. Morris, J.C. The Clinical Dementia Rating (CDR): current version and scoring rules. *Neurology* **43**, 2412-4 (1993).

7. Dooneief, G., Marder, K., Tang, M.X. & Stern, Y. The Clinical Dementia Rating scale: community-based validation of "profound' and "terminal' stages. *Neurology* **46**, 1746-9 (1996).

8. Braak, H. & Braak, E. Neuropathological stageing of Alzheimer-related changes. *Acta Neuropathol* **82**, 239-59 (1991).

9. Katsel, P., Tan, W. & Haroutunian, V. Gain in brain immunity in the oldest-old differentiates cognitively normal from demented individuals. *PLoS One* **4**, e7642 (2009).

10. Jonsson, T. *et al.* Variant of TREM2 associated with the risk of Alzheimer's disease. *N Engl J Med* **368**, 107-16 (2013).

11. Braak, H., Alafuzoff, I., Arzberger, T., Kretzschmar, H. & Del Tredici, K. Staging of Alzheimer disease-associated neurofibrillary pathology using paraffin sections and immunocytochemistry. *Acta Neuropathol* **112**, 389-404 (2006).

12. Birkett, P. *et al.* Executive function and genetic predisposition to schizophrenia--the Maudsley family study. *Am J Med Genet B Neuropsychiatr Genet* **147**, 285-93 (2008).

13. Wechsler, D. *WAIS-III WMS-III: Technical manual*, (Psychological Corporation, San Antonio, 1997).

14. Bechara, A., Damasio, H., Tranel, D. & Anderson, S.W. Dissociation Of working memory from decision making within the human prefrontal cortex. *J Neurosci* **18**, 428-37 (1998).

15. Fletcher, P.C. & Henson, R.N. Frontal lobes and human memory: insights from functional neuroimaging. *Brain* **124**, 849-81 (2001).

16. Owen, A.M., Downes, J.J., Sahakian, B.J., Polkey, C.E. & Robbins, T.W. Planning and spatial working memory following frontal lobe lesions in man. *Neuropsychologia* **28**, 1021-34 (1990).

17. Park, S.B. *et al.* Tryptophan depletion in normal volunteers produces selective impairments in learning and memory. *Neuropharmacology* **33**, 575-88 (1994).

**Supplement Table 1.** TaqMan gene expression assays used in the study

| Gene symbol | AB Assay ID | UniGene ID | Exon Boundary |
| --- | --- | --- | --- |
| *GUSB* | Hs00939627_m1 | Hs.255230 | 8 - 9 |
| *RPLPO* | Hs99999902_m1 | Hs.546285 | 3 - 3 |
| *PPIA* | Hs99999904_m1 | Hs.356331, Hs.598115 | 4 - 4 |
| *DRD1* | Hs00265245_s1 | Hs.2624 | 2 - 2 |

**Supplement Table 2.** Association of COGS SNPs with CDR. Only *P* < 0.05 are shown. Empirical *P* (*P_emp_*) is estimated based on 100,000 permutations/

| **SNP** | **CHR** | **BP** | **Gene Symbol** | **Gene Description** | **Allele** | ***β*** | **T stat** | ***P*** | ***P_emp_*** |
| --- | --- | --- | --- | --- | --- | --- | --- | --- | --- |
| **rs5326** | 5 | 174870196 | DRD1 | dopamine receptor D1 | A | 0.7419 | 3.325 | 0.0009325 | 0.004 |
| **rs7895098** | 10 | 87650378 | GRID1 | glutamate receptor, ionotropic, delta 1 | A | -0.4639 | -2.492 | 0.01294 | 0.0128 |
| **rs4934150** | 10 | 87865374 | GRID1 | glutamate receptor, ionotropic, delta 1 | G | -0.4583 | -2.355 | 0.0188 | 0.0192 |
| **rs6943659** | 7 | 86395926 | GRM3 | glutamate receptor, metabotropic 3 | C | 0.3962 | 2.245 | 0.02511 | 0.0254 |
| **rs1902667** | 10 | 87949239 | GRID1 | glutamate receptor, ionotropic, delta 1 | C | 0.3787 | 2.193 | 0.02862 | 0.0294 |
| **rs12266132** | 10 | 87823099 | GRID1 | glutamate receptor, ionotropic, delta 1 | T | 0.543 | 2.111 | 0.03517 | 0.0361 |
| **rs7001605** | 8 | 32347247 | NRG1 | neuregulin 1 | G | -0.3417 | -2.091 | 0.03687 | 0.0368 |
| **rs1896526** | 10 | 88069488 | GRID1 | glutamate receptor, ionotropic, delta 1 | G | 0.457 | 2.088 | 0.03716 | 0.0383 |
| **rs2068190** | 5 | 148007013 | HTR4 | 5-hydroxytryptamine (serotonin) receptor 4 | T | 0.3207 | 2.058 | 0.03998 | 0.0398 |
| **rs1011427** | 5 | 147987977 | HTR4 | 5-hydroxytryptamine (serotonin) receptor 4 | T | 0.5276 | 2.036 | 0.04213 | 0.042 |
| **rs12575544** | 11 | 112918985 | NCAM1 | neural cell adhesion molecule 1 | A | -0.3735 | -1.996 | 0.04637 | 0.0438 |
| **rs1322783** | 1 | 231929075 | DISC1 | disrupted in schizophrenia 1 | T | 0.4547 | 1.964 | 0.04991 | 0.052 |

**Supplement Table 3.** Demographics of the *DRD1* rs5326 genotype groups for the LOGOS cohort

|  | | | | |
| --- | --- | --- | --- | --- |
|  | **GG** | **GA** | **AA** | ***P*** |
| **Sample size** | 1099 | 341 | 30 |  |
| **Age, years^a^** | 22.3±3.6 | 21.4±3.2 | 21.8±3.3 | <0.0001 |
| **IQ, Raven’s raw score^a^** | 49.2±7.4 | 48.8±7.7 | 49.0±6.6 | 0.631 |
| **Smokers/Non-smokers^b^** | 507/592 | 131/210 | 11/19 | 0.031 |
| **Smokers: Cigarettes/day^a^** | 7.5±10.0 | 5.6±8.3 | 5.2±7.6 | 0.012 |
|  |  |  |  |  |
| ^a^Kruskal-Wallis comparison, ^b^x^2^ comparison | |  |  |  |
| Values represent mean ± SD |  |  |  |  |

**Supplement Table 4. Principal component analysis factor loadings of the cognitive variables for the LOGOS cohort**

| Task | Item | 1. Declarative Memory: Eigenvalue: 4.942, variance explained: 22.464% | 2. Problem Solving: Eigenvalue: 2.201, variance explained: 10.004% | 3. Sustained Attention: Eigenvalue: 1.726, variance explained: 7.845% | 4. Inattention: Eigenvalue: 1.696, variance explained: 7.709% | 5. Set-Shifting: Eigenvalue: 1.497, variance explained: 6.804% | 6. Emotional Decision Making: Eigenvalue: 1.392, variance explained: 6.325% | 7. Strategy Formation: Eigenvalue: 1.276, variance explained: 5.798% | 8. Working Memory: Eigenvalue: 1.218, variance explained: 5.536% | 9. Planning: Eigenvalue: 1.117, variance explained: 5.076% |
| --- | --- | --- | --- | --- | --- | --- | --- | --- | --- | --- |
| WL | Correct responses: immediate | 0.791 |  |  |  |  |  |  |  |  |
|  | Correct responses: short-delay | 0.905 |  |  |  |  |  |  |  |  |
|  | Correct responses: long-delay | 0.894 |  |  |  |  |  |  |  |  |
|  | Intrusions: immediate |  |  |  | 0.722 |  |  |  |  |  |
|  | Intrusions: short-delay |  |  |  | 0.853 |  |  |  |  |  |
|  | Intrusions: long-delay |  |  |  | 0.842 |  |  |  |  |  |
| SOC | Problems |  | 0.896 |  |  |  |  |  |  |  |
|  | Mean moves |  | -0.894 |  |  |  |  |  |  |  |
|  | Mean Initial Time Thinking |  |  |  |  |  |  |  |  | 0.786 |
|  | Mean Subsequent Time Thinking |  |  |  |  |  |  |  |  | 0.672 |
| RVIP | Correct rejections |  |  | 0.931 |  |  |  |  |  |  |
|  | Correct responses |  |  | 0.931 |  |  |  |  |  |  |
| WCST | Completed categories |  |  |  |  | 0.95 |  |  |  |  |
|  | Total errors |  |  |  |  | -0.932 |  |  |  |  |
| IGT | Advantageous cards |  |  |  |  |  | 0.943 |  |  |  |
|  | Total money won |  |  |  |  |  | 0.953 |  |  |  |
| SWM | Between errors |  |  |  |  |  |  | 0.781 |  |  |
|  | Within errors |  |  |  |  |  |  | 0.814 |  |  |
|  | Strategy score |  |  |  |  |  |  | 0.571 |  |  |
| N-back | Correct 1-back |  |  |  |  |  |  |  | 0.814 |  |
|  | Correct 2-back |  |  |  |  |  |  |  | 0.662 |  |
|  | Correct 3-back |  |  |  |  |  |  |  | 0.588 |  |
|  |  |  |  |  |  |  |  |  |  |  |
| ANCOVA | *F(2,1465)* | 1.654 | 0.073 | 1.034 | 0.704 | 0.161 | 0.577 | 1.018 | 0.022 | **5.847** |
|  | *η^2^* | 0.002 | <0.001 | 0.001 | 0.001 | <0.001 | 0.001 | 0.001 | <0.001 | **0.008** |
|  | *P* | 0.192 | 0.93 | 0.356 | 0.495 | 0.851 | 0.562 | 0.362 | 0.978 | **0.003** |

IGT: Iowa Gambling Task; N-back: N-Back Sequential Letter Task; RVIP: Rapid Visual Information Processing; SoC: Stocking of Cambridge; SWM: Spatial Working memory; WCST: Wisconsin Card Sorting Test; WL: Word Lists task

Bold fonts represents association that survived Bonferroni correction (0.05/9 = 0.006)

**Supplement Table 5.** Demographics of the *DRD1* rs5326 genotype groups for the human postmortem - gene expression cohort

|  | | | | |
| --- | --- | --- | --- | --- |
|  | **GG** | **GA** | **AA** | ***P*** |
| **Sample size** | 77 | 29 | 3 |  |
| **Age, years^a^** | 68.7±17.4 | 72.7±18.9 | 92±2.6 | 0.062 |
| **Sex (Male/Female)^b^** | 46/31 | 18/11 | 2/1 | 0.953 |
| **PMI, hours^a^** | 15.7±10.0 | 14.6±10.6 | 8.1±10.1 | 0.419 |
| **RIN^a^** | 6.5±1.2 | 6.0±1.7 | 5.2±1.5 | 0.022 |
|  |  |  |  |  |
| ^a^ANOVA comparison, ^b^x2 comparison | | |  |  |
| Values represent mean ± SD | |  |  |  |
